# Application of an Antioxidant Response Element–Nuclear Factor Erythroid 2 Luciferase Assay for Assessing the Skin Sensitization Potential of Agrochemicals

**DOI:** 10.3390/toxics11120973

**Published:** 2023-11-30

**Authors:** Ji-Yeon Yang, Soo-Jin Park, Ji-Young Shin, Jeong-Hyun Lim, Si Young Yang, Geun-Hwan Gil, So-Hye Hong

**Affiliations:** Toxicity and Risk Assessment Division, Department of Agro-Food Safety & Crop Protection, National Institute of Agricultural Sciences, Rural Development Administration, Wanju 55365, Jeollabuk-do, Republic of Korea; jiyeon0527@korea.kr (J.-Y.Y.); jiny0451@korea.kr (S.-J.P.); shinji00@korea.kr (J.-Y.S.); jhlim0531@korea.kr (J.-H.L.); siyoungyang@korea.kr (S.Y.Y.); ghgil@korea.kr (G.-H.G.)

**Keywords:** skin sensitization, agrochemicals, alternative methods, antioxidant response element, nuclear factor erythroid 2 luciferase assay, adverse outcome pathway

## Abstract

The skin sensitization potential of agrochemicals can be assessed using laboratory methods such as the keratinocyte activation assay so that their use in regulatory toxicology might replace experimental animal testing. Here, we evaluated the skin sensitization potential of 11 agrochemicals by using an antioxidant response element–nuclear factor erythroid 2 luciferase assay in KeratinoSens and LuSens cells and applying a skin sensitization adverse outcome pathway (AOP). The KeratinoSens and LuSens assays consistently evaluated the skin sensitization potential of 10/11 agrochemicals with reference to animal testing databases. Benomyl, pretilachlor, fluazinam, terbufos, butachlor, and carbosulfan were correctly detected as sensitizers, and glufosinate ammonium, oxiadiazon, tebuconazole, and etofenprox were correctly detected as non-sensitizers. For diazinon, the skin sensitizing potential was positive in the KeratinoSens assay but not in the LuSens assay. These results suggest that the evaluation of in vitro skin sensitization using the AOP mechanism can be applied to assess active agrochemicals.

## 1. Introduction

The assessment of the skin sensitization potential is a part of the chemical hazard evaluation of agrochemicals, cosmetics, and industrial chemicals. In the registration process of agrochemicals, the evaluation of the skin sensitization potential is required for the global registration of active ingredients to ensure the safety of agricultural workers and operators [1]. Traditionally, the skin sensitization hazard of an agrochemical is assessed via animal testing such as the guinea pig maximization test (GPMT, OECD TG 406), Buehler test (OECD TG 406) [2], and mouse local lymph node assay (LLNA, OECD TGs 429, 442A, 442B) [3,4,5]. Owing to the increasing awareness of the ethical aspects of animal testing, alternative laboratory methods based on sensitization mechanisms not requiring experimental animal testing have been actively pursued.

According to the OECD adverse outcome pathway (AOP) for skin sensitization [6], non-animal tests focus on three key events (KEs): KE1, protein binding; KE2, keratinocyte activation; and KE3, dendritic cell activation. KEs are detected using three methods, namely the direct peptide reactivity assay (DPRA), the amino acid derivative reactivity assay (ADRA), and the kinetic direct peptide reactivity assay (kDPRA) (OECD TG 442C) [7]. The first assesses the chemical reactivity to skin proteins; the second evaluates keratinocyte activation with the KeratinoSens and LuSens assays (OECD TG 442D) [8]; and the third evaluates dendritic cell activation using the human cell line activation test (h-CLAT), the U937 cell line activation test (U-SENS), the interleukin-8 reporter gene assay (IL-8 Luc assay), or the genomic allergen rapid detection (GARD) for the assessment of skin sensitizers (GARD skin) (OECD TG 442E) [9]. KE2 in skin sensitization is the induction of cell-protective genetic pathways in keratinocytes, the front-line epidermic cells exposed to skin sensitizers. The nuclear factor erythroid 2 (Nrf2)–Kelch-like ECH-associated protein 1 (Keap1) pathway plays a vital role in skin sensitization [10,11]. The sensor protein Keap1 contains highly reactive cysteine residues. When a sensitizer enters the cytosol, the covalent modification of cysteine residues in Keap1 leads to its dissociation from the transcription factor Nrf2 [12]. Free Nrf2 accumulates in the nucleus, forming heterodimers with other molecules [12]. This complex binds to the antioxidant response element (ARE) in the promoter sequence [4]. Based on the principle of this pathway, chemicals known as sensitizers or non-sensitizers are tested for luciferase induction in KeratinoSens and LuSens cell lines to increase the predictive capacity of parameters such as accuracy, sensitivity, and specificity by a keratinocyte activation.

A single point of in vitro non-animal test is insufficient to predict the skin sensitization potential of chemicals because it provides limited information on the mechanisms and does not achieve 100% accuracy of in vivo animal data. This limitation can be overcome via integrated approaches to testing and assessment (IATA), which need to match numerous requirements and might not be considered acceptable by the OECD Mutual Acceptance of Data Agreement [13]. However, defined approaches (DAs) consisting of the integrated test results can be applied, which is covered by the Acceptance of Data agreement [14]. The document published by the OECD [15,16] describes reporting guidelines of defined approaches and individual information within several integrated testing strategies for identifying skin sensitization hazards. Following the issuing of OECD no. 497 on defined approaches for skin sensitization [16], there is a need for globally harmonized testing of agrochemicals using individual in chemico, in vitro, and in silico test methods and DAs [17]. Guideline 497 includes three specific DAs. The “2 out of 3” (2o3) DA depends on two harmonized results for sensitizing hazard classification from three non-animal tests [16]. While the LLNA data are approximately 80% accurate compared to the human data, the 2o3 DA is approximately 90% accurate compared to the human data [18,19]. Therefore, in the 2o3 DA, the results of two non-animal tests are considered sufficient for the final prediction.

The protein reactivity potential of agrochemicals has been previously evaluated using the DPRA [20]. However, a single method may not be able to achieve high predictivity owing to the complexity of skin sensitization mechanisms. Instead, the ARE–Nrf2 luciferase assay using KeratinoSens and LuSens (KE2) can provide sufficient information on protein binding. We reasoned that the binary test battery with KeratinoSens and LuSens could provide sufficient information on keratinocyte activation (KE2 triggered by KE1) and might lead to a mechanism based on AOP as part of an approach for predicting skin sensitization hazard [21]. In the present study, we evaluated the skin sensitization potential of agrochemicals using an ARE–Nrf2 luciferase assay in KeratinoSens and LuSens cells and compared the results with in vivo data. The predictive capacity of a binary test battery with KeratinoSens and LuSens cells was examined with the database of agrochemicals and compared with the current two out of two DA consisting of DPRA, KeratinoSens, and LuSens.

## 2. Materials and Methods

### 2.1. Test Substances

As part of the initial experiments to validate the technical proficiency of the laboratory, we evaluated the skin sensitization potential of the OECD TG 442D proficiency test substances [8] (ARE–Nrf2 luciferase test method) and compared the in-house results with in vivo assays (guinea pigs), and then published KeratinoSens and LuSens assay results. All compounds and agrochemicals were purchased from Sigma-Aldrich (St. Louis, MO, USA). All compounds were dissolved in dimethyl sulfoxide (DMSO, purity > 99.9%, Sigma-Aldrich) to prepare a working solution. The final concentration of DMSO was 0.1% in both vehicle control and treatment groups. In our study, active ingredients of agrochemicals were assessed for skin sensitization potential using the KeratinoSens and LuSens assays (Table 1).

### 2.2. KeratinoSens Cell Culture

KeratinoSens cells derived from transgenic human keratinocytes were provided by Givaudan Suisse SA (Vernier, Switzerland). Cells were cultured in Dulbecco’s Modified Eagle Medium (DMEM; Gibco, Carlsbad, CA, USA) supplemented with 10% fetal bovine serum (FBS; Gibco, Calsbad, CA, USA) and 0.5 mg/mL geneticin (Sigma-Aldrich, St. Louis, MO, USA) at 37 °C with 5% CO_2_ in a humidified atmosphere. Cells were sub-cultured every 2–3 days at 85–90% confluence for a maximum of 25 passages.

### 2.3. LuSens Cell Culture

LuSens cells derived from human keratinocytes were provided by BASF (Ludwigshafen, Germany) and cultured in DMEM supplemented with 10% FBS and 1% penicillin–streptomycin (Gibco, Calsbad, CA, USA). After 24 h, the media was replaced with DMEM containing 10% FBS, 1% penicillin–streptomycin, and 0.005% puromycin (Sigma-Aldrich, St. Louis, MO, USA) at 37 °C with 5% CO_2_ in a humidified atmosphere. Cells were sub-cultured three times a week via trypsinization using 0.25% trypsin–ethylenediaminetetraacetic acid solution (Gibco) at 85–90% confluence for a maximum of 15 passages.

### 2.4. KeratinoSens Assay Method

The KeratinoSens assay was performed following standard protocols described by OECD TG 442D [8]. KeratinoSens cells were seeded in 96-well plates at a density of 1.0 × 10^4^ cells/well. Each substance was tested in the range of 0.98–2000 μM in three wells. Additionally, the plates contained a vehicle control (DMSO) in six wells, positive control (cinnamic aldehyde at five different concentrations) in five wells, and blank control (no cells) in one well. In repeated experiments, cell viability was measured three times using a thiazolyl blue tetrazolium bromide (MTT) assay (Sigma-Aldrich). Luciferase activity was measured according to a standard protocol (One-Glo Luciferase Assay System Kit; Promega, Madison, WI, USA) under the same conditions as that of the MTT assay. The luminescence intensity of each sample was measured using a microplate reader (Thermo Fisher Scientific, Waltham, USA). Test substances were considered positive in the KeratinoSens assay if the following criteria were fulfilled: (1) I_max_ was ≥1.5 fold and statistically significant as compared with that of the vehicle control; (2) cell viability was higher than 70% at the lowest concentration in which the luciferase induction is >1.5-fold; (3) EC_1.5_ value was <1000 μM; and (4) a dose-dependent increase in the luciferase activity was induced.

### 2.5. LuSens Assay Method

The LuSens assay was conducted according to standard protocols published by OECD TG 442D [8] and consisted of two experimental phases. Briefly, LuSens cells were seeded in 96-well plates at a density of 1 × 10^4^ cells/well and incubated for 24 h. The test substances were applied in the range of 0.98–2000 μM. Additionally, the plates contained ethylene glycol dimethylacrylate as a positive control, lactic acid as a negative control, and DMSO as vehicle control. After 48 h, cell viability was measured using an MTT assay. The Luciferase assay was conducted under the same conditions as that of the MTT assay. After 24 h, each test substance was added at six concentrations (CV_75_/2.07, CV_75_/1.73, CV_75_/1.44, CV_75_/1.2, CV_75_, and 1.2 × CV_75_) in triplicates. After treatment, ARE–Nrf2 activation was measured by luciferase assay (Steady-Glo Luciferase Assay; Promega) using a microplate reader (Thermo Fisher Scientific, Waltham, MA, USA). Test substances were predicted as positive if the following conditions were fulfilled: (1) induction of luciferase activity was >1.5 fold when compared with that of the vehicle control for at least two consecutive testing concentrations; (2) luciferase induction was statistically significant; and (3) at least three testing concentrations were noncytotoxic.

### 2.6. Statistical Analysis

The statistical analysis was conducted using Prism v.5.0 (La Jolla, CA, USA), and each group was compared using a two-tailed paired Student’s *t*-test. A *p*-value < 0.05 was considered statistically significant. The predictive capability of ARE–Nrf2 luciferase assay for test substances was calculated according to Cooper statistics [30] for sensitivity, specificity, and accuracy. Sensitivity was defined as the fraction of sensitizers that were identified as positive. Specificity was defined as the fraction of non-sensitizers identified as negative. Accuracy was the fraction of accurate predictions.

## 3. Results

### 3.1. Laboratory Proficiency of the KeratinoSens Assay

In a proficiency test to assess the technical proficiency and reproducibility of the KeratinoSens assay, 10 proficiency substances were correctly detected as sensitizers (ethylene glycol dimethacrylate, cinnamyl alcohol, 2-mercaptobenzothiazole, 4-methylaminophenol sulfate, methyldibromoglutaronitrile, and 2,4-dinitro-chlorobenzene) or non-sensitizers (salicylic acid, lactic acid, glycerol, and isopropanol) by the KeratinoSens cell line. (Table 2). The EC_1.5_ and IC_50_ values all the proficient substances, but cinnamyl alcohol fell within the reference ranges provided by OECD TG 442D [8]. Therefore, the technical proficiency of the laboratory was considered good.

### 3.2. Laboratory Proficiency with the LuSens Assay

We also assessed the technical proficiency and reproducibility of the LuSens assay the ten proficiency substances listed in the OECD test guidelines (Table 3), including six skin sensitizers and four non-sensitizers. The results for all but cinnamyl alcohol were consistent with the OECD test guidelines and the published literature (Table 3). The sensitizers exhibited dose-dependent increases in luciferase activity above the threshold of 1.5-fold at concentrations below 1.2 × CV_75_. The non-sensitizers did not induce luciferase activity or induced the responses only at cytotoxic concentrations at which cell viability was >75%. These results confirm the technical proficiency of the laboratory in conducting the LuSens assay.

### 3.3. Evaluation of Agrochemicals Using the ARE-Nrf2 Luciferase Assay

**Figure 1:**
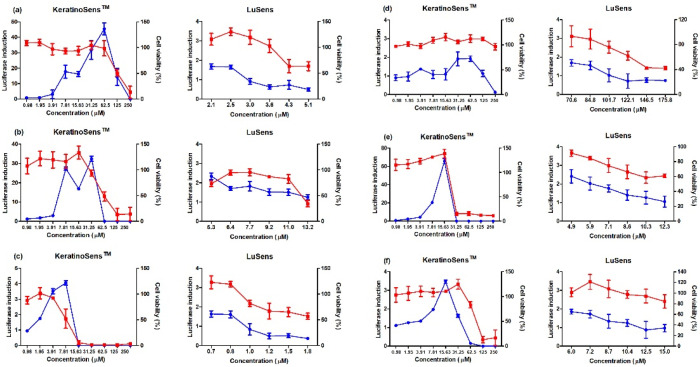
Results of luciferase induction (circles; blue color) and cell viability (squares; red color) for sensitizer agrochemicals using the ARE-Nrf2 luciferase assay; induction of luciferase activity was more than 1.5 fold, and cell viability was over 70%, respectively: (**a**) benomyl, (**b**) pretilachlor, (**c**) fluaziman, (**d**) terbufos, (**e**) butachlor, and (**f**) carbosulfan. Positive control (cinnamic aldehyde, 4–64 μM against KeratinoSens^TM^ cells and ethylene glycol dimethylacrylate 120 μM against LuSens cells) was tested. Each group was compared with the vehicle control. All of the experiments were repeated three times. Data are expressed as mean ± standard deviation values (*n* = 3).

**Figure 2:**
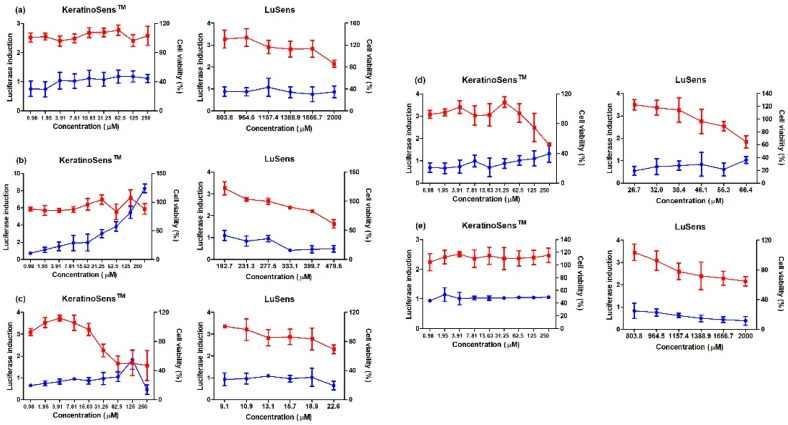
Results of luciferase induction (circles; blue color) and cell viability (squares; red color) for non-sensitizer agrochemicals using the ARE-Nrf2 luciferase assay; induction of luciferase activity was more than 1.5 fold, and cell viability was over 70%, respectively: (**a**) glufosinate ammonium, (**b**) diazinon, (**c**) oxadiazon, (**d**) tebuconazole, and (**e**) etofenprox. Positive control (cinnamic aldehyde, 4–64 μM against KeratinoSens^TM^ cells and ethylene glycol dimethylacrylate 120 μM against LuSens cells) was tested. Each group was compared with the vehicle control. All of the experiments were repeated three times. Data are expressed as mean ± standard deviation values (*n* = 3).

To evaluate the AOP KE2 relevant to keratinocyte activation, 11 agrochemicals were assessed for skin sensitization potential using the KeratinoSens and LuSens assays; the induction of luciferase activity had to be more 1.5 fold, with cell viability was over 70%, respectively. (Figure 1 and Figure 2) Among the selected agrochemicals, six were reported as sensitizers (benomyl, pretilachlor, fluazinam, terbufos, butachlor, and carbosulfan), and five as non-sensitizers (glufosinate ammonium, diazinon, oxadiazon, tebuconazole, and etofenprox) in published in vivo assays [22,23,24,25,26,27,28,29]. In the KeratinoSens assay, the EC_1.5_ values for benomyl, pretilachlor, fluazinam, terbufos, butachlor, and carbosulfan were 3.33, 1.39, 1.65, 2.48, 1.41, and 4.86 μM, respectively. In the LuSens assay, the EC_1.5_ values were <2.1, <5.3, <0.7, <70.6, <4.9, and <6.0 μM, respectively. The KeratinoSens assay determined the EC_1.5_ values for glufosinate ammonium, oxadiazon, tebuconazole, and etofenprox as >2000 μM, while the EC_1.5_ value of diazinon was 20.66. In the LuSens assay, the EC_1.5_ values were more than 1.2 × CV_75_ values, respectively. The induction values were <1.5. For diazinon, the skin sensitivity was positive in the KeratinoSens assay but negative in the LuSens assay.

### 3.4. Comparison of Prediction for Skin Sensitization

To confirm the predictive capacity of the KeratinoSens and LuSens assays, we compared the test results with the animal sensitization data from the literature using Cooper’s statistics (Table 4). The KeratinoSens assay correctly predicted 10 of the 11 agrochemicals, and the LuSens assay correctly predicted all the tested agrochemicals. In the KeratinoSens assay, diazinon was incorrectly rated as positive compared to the animal data. In addition, data obtained from the KeratinoSens and LuSens assays were compared with animal data from the literature. According to Kolle et al. [33], the borderline ranges from the official ring trials are 1.35 to 1.67 for the KeratinoSens and 1.28 to 1.76 for the LuSens. Via this analysis, the following predictivity values were calculated: sensitivity of 100% and 100%, specificity of 83.3% and 100%, and accuracy of 90.9% and 100% for the KeratinoSens and LuSens assays, respectively.

This approach predicted the skin sensitization potential based on two of three tests addressing protein reactivity (e.g., DPRA), keratinocyte ARE activation (e.g., KeratinoSens and LuSens), and dendritic cell activation (e.g., h-CLAT). Concordant results of the two tests determined whether a substance was a sensitizer [34]. When the KeratinoSens and LuSens assay results were used in combination with previously reported data for DPRA [20], the skin sensitization potential of all the agrochemicals, but diazinon was in accordance with animal data; the sensitivity, specificity, and accuracy were 100% (Table 4). In the case of diazinon, the skin sensitization potential using DPRA (KE1) and LuSens assay (KE2) was negative, while it was positive in the KeratinoSens assay (KE2). Therefore, we could not determine the skin sensitization potential of diazinon.

## 4. Discussion

The present study describes the results of agrochemical sensitization tests using the ARE–Nrf2 Luciferase Keratinosens and LuSens assays. The LuSens assay results were 100% consistent with those from the available in vivo databases but the result differed between the two tests for diazinon sensitization. An additional assay, such as h-CLAT addressing KE3, would be required to confirm its sensitization potential.

In a previous study, the same agrochemicals were tested using an in chemico Direct Peptide Reactivity Assay for Skin Sensitization [20]. This test performed less efficiently as it correctly predicted six pesticides as sensitizers (benomyl, butachlor, carbosulfan, fluazinam, pretilachlor, and terbufos) and four as non-sensitizers (diazinon, glufosinate ammonium, oxadiazon, and tebuconazole). However, relying on the results of a single KE test does not reliably detect skin sensitization [35].

Defined approaches utilize results from multiple non-animal information to achieve a predictive capacity for human skin sensitization potential equal to that of animal testing. For 10/11 agrochemicals, Keratinocyte (KE2) and Lusens reactions consistently detected sensitizers and non-sensitizers.

Another study using the in vitro KeratinoSens assay correctly predicted the skin sensitization potential of another eight agrochemicals when compared to in vivo data [36]: acetochlor, meptyldinocap, and triclopyr tested positive, and aminopyralid, clopyralid, florasulam, methoxyfenzide, and oxyfluoren tested negative. The ARE–Nrf2 luciferase assay suffers from the limitation that it depends on the chemical’s ability to react covalently with cysteine units. Chemicals that can be sensitized by other reaction mechanisms can fail to give positive responses in this assay [15]. However, our results demonstrated that both the KeratinoSens and LuSens assays can be used to identify the mechanism of keratinocyte activation.

Alternatives to animal testing are continuously being developed because of its limitations with respect to the differences between animals and humans and because of animal ethics. In the future, alternative animal testing will become an unavoidable tool in the field of regulatory toxicology. Furthermore, improving pesticide evaluation methods, such as mixed application and insoluble substance approaches, is necessary to reduce experimental animal testing.

## Figures and Tables

**Table 1:** Agrochemicals tested in the current study using the KeratinoSens^TM^ and the LuSens assays.

Agrochemicals	CAS RN	MW	Classification	Classification from Published In Vivo Data	
				Results	Literature
Glufosinate ammonium	77182-82-2	181	Herbicide	Non-sensitizer	[22]
Diazinon	333-41-5	304	Insecticide	Non-sensitizer	[23]
Oxadiazon	19666-30-9	345	Herbicide	Non-sensitizer	[24]
Tebuconazole	107534-96-3	308	Fungicide	Non-sensitizer	[25]
Etofenprox	80844-07-1	376	Insecticide	Non-sensitizer	[25]
Benomyl	17804-35-2	290	Fungicide	Sensitizer	[26]
Pretilachlor	51218-49-6	312	Herbicide	Sensitizer	[27]
Fluazinam	79622-59-6	465	Fungicide	Sensitizer	[28]
Terbufos	13071-79-9	288	Insecticide	Sensitizer	[29]
Butachlor	23184-66-9	312	Herbicide	Sensitizer	[27]
Carbosulfan	55285-14-8	381	Insecticide	Sensitizer	[29]

**Table 2:** Proficiency of the KeratinoSens^TM^ assay.

Proficiency Substances	CAS RN	Physical Form	In Vivo Prediction ^1^	EC_1.5_ (μM) Reference Range ^2^	IC_50_ (μM) Reference Range ^2^	KeratinoSens^TM^ Assay		
						EC_1.5_ (μM)	IC_50_ (μM)	Results
Isopropanol	67-63-0	Liquid	Non-sensitizer	>1000	>1000	>2000	>2000	Negative
Salicylic acid	69-72-7	Solid	Non-sensitizer	>1000	>1000	>2000	>2000	Negative
Lactic acid	50-21-5	Liquid	Non-sensitizer	>1000	>1000	>2000	>2000	Negative
Glycerol	56-81-5	Liquid	Non-sensitizer	>1000	>1000	>2000	>2000	Negative
Cinnamyl alcohol	104-54-1	Solid	Weak sensitizer	25–175	>1000	179.72	>2000	Positive
Ethylene glycol dimethacrylate	97-90-5	Liquid	Weak sensitizer	5–125	>500	18.25	1567.32	Positive
2-Mercapto benzothiazole	149-30-4	Solid	Moderate sensitizer	25–250	>500	202.31	699.85	Positive
Methyldibromo glutaronitrile	35691-65-7	Solid	Strong sensitizer	<20	20–100	3.02	25.16	Positive
4-Methyl aminophenol sulfate	55-55-0	Solid	Strong sensitizer	<12.5	20–100	2.99	30.87	Positive
2,4-Dinitro-chlorobenzene	97-00-7	Solid	Extreme sensitizer	<12.5	5–20	2.03	12.18	Positive

^1^ The in vivo hazard and potency predictions are based on LLNA data [31]. The in vivo potency is derived using the criteria proposed by ECETOC [32]. ^2^ Information on the reference range was provided in OECD test guideline 442D.

**Table 3:** Proficiency of the LuSens assay.

Proficiency Substances	CAS RN	Physical Form	In Vivo Prediction ^1^	EC_1.5_ (μM) Reference Range ^2^	CV_75_ (μM) Reference Range ^2^	LuSens Assay		
						EC_1.5_ (μM)	CV_75_ (μM)	Results
Salicylic acid	69-72-7	Solid	Non-sensitizer	>1000	>2000	>2000	>2000	Negative
Glycerol	56-81-5	Liquid	Non-sensitizer	>1000	>2000	>2000	>2000	Negative
Isopropanol	67-63-0	Liquid	Non-sensitizer	>1000	>2000	>2000	>2000	Negative
Sulfanilamide	63-74-1	Solid	Non-sensitizer	>1000	>2000	>2000	>2000	Negative
Eugenol	97-53-0	Liquid	Weak sensitizer	<500	<1000	437.5	453.1	Positive
Cinnamyl alcohol	104-54-1	Solid	Weak sensitizer	<170	>420	433.0	622.3	Positive
2-Mercapto benzothiazole	149-30-4	Solid	Moderate sensitizer	<800	<2000	211.3	216.3	Positive
4-Methyl aminophenol sulfate	55-55-0	Solid	Strong sensitizer	<30	<50	<8.4	17.5	Positive
Methyldibromo glutaronitrile	35691-65-7	Solid	Strong sensitizer	<25	<50	<5.5	11.3	Positive
2,4-Dinitro-chlorobenzene	97-00-7	Solid	Extreme sensitizer	<5	<10	<1.5	3.2	Positive

^1^ The in vivo hazard and potency predictions are based on LLNA data [33]. The in vivo potency is derived using the criteria proposed by ECETOC [34]. ^2^ Information on the reference range was provided in OECD test guideline 442D.

**Table 4:** Comparison of prediction of the skin sensitization among KeratinoSens^TM^ and the LuSens for the agrochemicals.

Agrochemicals	Animal Data	DPRA ^1^	KeratinoSens^TM^	LuSens	Final Prediction
Glufosinate ammonium	Non-sensitizer	− ^2^	−	−	Non-sensitizer
Diazinon	Non-sensitizer	−	+	−	ND ^4^
Oxadiazon	Non-sensitizer	−	−	−	Non-sensitizer
Tebuconazole	Non-sensitizer	−	−	−	Non-sensitizer
Etofenprox	Non-sensitizer	−	−	−	Non-sensitizer
Benomyl	Sensitizer	+ ^3^	+	+	Sensitizer
Pretilachlor	Sensitizer	+	+	+	Sensitizer
Fluazinam	Sensitizer	+	+	+	Sensitizer
Terbufos	Sensitizer	+	+	+	Sensitizer
Butachlor	Sensitizer	+	+	+	Sensitizer
Carbosulfan	Sensitizer	+	+	+	Sensitizer
Sensitivity (%)		100	100	100	
Specificity (%)		100	83.3	100	
Accuracy (%)		100	90.9	100	

^1^ Data were obtained from Lee et al. [20]. ^2^ Negative/non-sensitizer predictions. ^3^ Positive/sensitizer prediction. ^4^ Not determined.

## Data Availability

The original contributions presented in the study are included in the article; further inquiries can be directed to the corresponding authors.
